# Homelessness and Risk of End-Stage Kidney Disease and Death in Veterans With Chronic Kidney Disease

**DOI:** 10.1001/jamanetworkopen.2024.31973

**Published:** 2024-09-10

**Authors:** Alain K. Koyama, Robert Nee, Wei Yu, Devasmita Choudhury, Fei Heng, Alfred K. Cheung, Monique E. Cho, Keith C. Norris, Guofen Yan

**Affiliations:** 1 Division of Diabetes Translation, Centers for Disease Control and Prevention, Atlanta, Georgia; 2 Walter Reed National Military Medical Center, Uniformed Services University, Bethesda, Maryland; 3 Department of Public Health Sciences, University of Virginia School of Medicine, Charlottesville; 4 Virginia-Tech Carilion School of Medicine Medical Center, Roanoke; 5 Salem Veterans Affairs Healthcare System, Salem, Virginia; 6 Department of Mathematics and Statistics, University of Florida, Jacksonville; 7 Division of Nephrology and Hypertension, University of Utah, Salt Lake City; 8 VA Salt Lake City Healthcare System, Salt Lake City, Utah; 9 Division of General Internal Medicine and Health Services Research, University of California Los Angeles, David Geffen School of Medicine, Los Angeles

## Abstract

**Importance:**

Adults experiencing homelessness in the US face numerous challenges, including the management of chronic kidney disease (CKD). The extent of a potentially greater risk of adverse health outcomes in the population with CKD experiencing homelessness has not been adequately explored.

**Objective:**

To evaluate the association between a history of homelessness and the risk of end-stage kidney disease (ESKD) and death among veterans with incident CKD.

**Design, Setting, and Participants:**

This retrospective cohort study was conducted between January 1, 2005, and December 31, 2017. Participants included veterans aged 18 years and older with incident stage 3 to 5 CKD utilizing the Veterans Health Administration health care network in the US. Patients were followed-up through December 31, 2018, for the occurrence of ESKD and death. Analyses were performed from September 2022 to October 2023.

**Exposure:**

History of homelessness, based on utilization of homeless services in the Veterans Health Administration or *International Classification of Diseases, Ninth Revision* or *International Statistical Classification of Diseases and Related Health Problems, Tenth Revision* codes. Homelessness was measured during the 2-year baseline period prior to the index date of incident CKD.

**Main Outcomes and Measures:**

The primary outcomes were ESKD, based on initiation of kidney replacement therapy, and all-cause death. Adjusted hazard ratios (HRs) were calculated to compare veterans with a history of homelessness with those without a history of homelessness.

**Results:**

Among 836 361 veterans, the largest proportion were aged 65 to 74 years (274 371 veterans [32.8%]) or 75 to 84 years (270 890 veterans [32.4%]), and 809 584 (96.8%) were male. A total of 26 037 veterans (3.1%) developed ESKD, and 359 991 (43.0%) died. Compared with veterans who had not experienced homelessness, those with a history of homelessness showed a significantly greater risk of ESKD (adjusted HR, 1.15; 95% CI, 1.10-1.20). A greater risk of all-cause death was also observed (HR, 1.48; 95% CI, 1.46-1.50). After further adjustment for body mass index, comorbidities, and medication use, results were attenuated for all-cause death (HR, 1.09; 95% CI, 1.07-1.11) and were no longer significant for ESKD (HR, 1.04; 95% CI, 0.99-1.09).

**Conclusions and Relevance:**

In this cohort study of veterans with incident stage 3 to 5 CKD, a history of homelessness was significantly associated with a greater risk of ESKD and death, underscoring the role of housing as a social determinant of health.

## Key Points

**Question:**

Is a history of homelessness associated with the risk of end-stage kidney disease (ESKD) or death among US veterans with incident stage 3 to 5 chronic kidney disease?

**Findings:**

In this cohort study of 836 361 veterans, a history of homelessness was significantly associated with a greater risk of both ESKD and all-cause death. The findings were likely explained by a greater comorbidity burden among veterans with a history of homelessness.

**Meaning:**

These findings suggest that among veterans with incident stage 3 to 5 chronic kidney disease, lack of housing may be a major determinant of adverse health outcomes.

## Teaser

This cohort study examines the association between a history of homelessness and the risk of end-stage kidney disease and death among US veterans with incident chronic kidney disease.

## Introduction

On any given day in 2023, approximately 653 000 individuals in the US were experiencing homelessness, with 4 in 10 residing in unsheltered locations.^1^ Experiencing homelessness can lead to a variety of adverse health outcomes, including an increased risk of falls, infection, cardiovascular disease, substance use disorder, and death.^2^ Chronic kidney disease (CKD) often requires intensive management to limit disease progression and systemic complications through lifestyle modification and/or pharmaceutical treatment. Given the social and economic challenges of homelessness, disease management is expected to be insufficient. Therefore, CKD complications can be of major health consequence among people experiencing homelessness.

Few prior studies have evaluated the risk of end-stage kidney disease (ESKD) or death among populations with CKD experiencing homelessness or with a history of homelessness in the US.^3,4^ In 2 studies^3,4^ of approximately 15 000 adults in San Francisco, California, with prevalent CKD (estimated by the Modification of Diet in Renal Disease study equation), experiencing homelessness was associated with a greater risk of ESKD and death. One recent study^5^ examined outcomes among veterans with ESKD receiving dialysis and found that unstable housing experienced within 3 years prior to starting dialysis was associated with an increased risk of all-cause mortality. Outside the US, the few studies^6,7^ that have investigated adults with CKD experiencing homelessness are limited to cross-sectional descriptive analyses. Within the US, veterans in particular may be at greater risk of homelessness; there are approximately 20 per 10 000 veterans experiencing homelessness compared with 18 per 10 000 individuals in the general population.^1^ In addition, the veteran population may provide practical and unique insight into CKD outcomes among adults experiencing homelessness, given their greater comorbidity burden compared with nonveteran adults experiencing homelessness^8,9^ and veterans’ access to the Veterans Health Administration (VHA), a nationwide health care system from which comprehensive data can be retrieved. To our knowledge, prior studies have not investigated outcomes among veterans who have experienced homelessness over the life course of CKD in the US. We, therefore, evaluated in a population of veterans with incident CKD whether a history of homelessness was associated with risk of ESKD and death, with the hypothesis that a greater risk of ESKD or all-cause mortality would be found among veterans with a history of homelessness. We also aimed to evaluate how these associations may differ among subgroups of age, sex, and race and ethnicity.

## Methods

Institutional review board approval was obtained at the University of Virginia and the Salem Veterans Affairs Medical Center, and the study was conducted in accordance with the Declaration of Helsinki.^10^ Informed consent was waived because the study only comprised secondary analysis of deidentified data, in accordance with 45 CFR §46. This study followed the Strengthening the Reporting of Observational Studies in Epidemiology (STROBE) reporting guidelines.

### Study Population

This study comprises veterans in the VHA, an integrated health care network providing primary care, specialized care, and other medical and social support services to more than 9 million veterans in the US at approximately 1300 facilities nationwide. At baseline, veterans were aged 18 years and older with incident CKD stages 3 to 5 between January 1, 2005, and December 31, 2017. The date of incident CKD, serving as the index date, was defined as the second of 2 estimated glomerular filtration rate (eGFR) values less than 60 mL/min/1.73 m^2^ measured at least 3 months apart, and for the first time over the study period. The eGFR was calculated using the 2021 CKD Epidemiology Collaboration creatinine equation that excludes race as a factor in the equation.^11^ Serum creatinine measurements obtained during inpatient acute care were excluded to avoid episodes of acute and reversible kidney injury.

Only veterans who were regular users of the VHA were included in this cohort. Regular users were defined a priori as VHA enrollees who fulfilled 2 criteria: (1) utilized VHA services that were documented in annual utilization files for the year of the index date and the 2 preceding calendar years; and (2) had at least 1 outpatient visit during the 2 years before the first of 2 eGFR values less than 60 mL/min/1.73 m^2^. These criteria were selected to maximize the validity of the index date in representing the veterans’ first lifetime occurrence of CKD. Veterans were excluded if they had a diagnosis of ESKD (defined as the initiation of kidney replacement therapy) before the index date.

**Figure 1.**
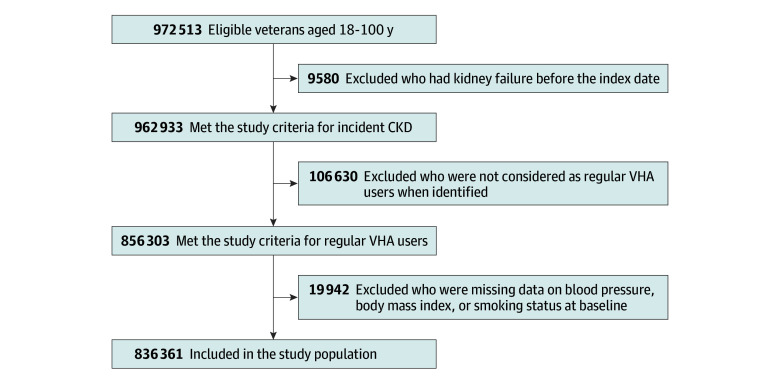
Flowchart of Cohort Construction To be eligible, veterans had to have the first clinical presentation of chronic kidney disease (CKD), defined as 2 estimated glomerular filtration rate (eGFR) values less than 60 mL/min/1.73 m^2^, for more than 3 months, identified in the Veterans Health Administration (VHA) database between January 1, 2005, and December 31, 2017, and were in the VHA for at least 2 years before their first eGFR value less than 60 mL/min/1.73 m^2^. The date of the second eGFR value served as the index date of CKD onset.

All laboratory test results were obtained from the VHA Corporate Data Warehouse (CDW). Because the cohort has been linked to Centers for Medicare & Medicaid Services (CMS) data, comorbidity data were based on records from both the VHA CDW and CMS Medicare Part A and Part B. A flowchart for the cohort construction is presented in Figure 1.

### Study Variables

The outcomes of interest were incident ESKD and death, which were analyzed as separate outcomes. ESKD was defined as the initiation of kidney replacement therapy (dialysis or kidney transplant), documented by linking the cohort to the US Renal Data System. The death outcome included all deaths, regardless of occurrence of ESKD, which was based on data from the VHA Vital Status File and VHA CDW Patient Domain. Follow-up time for death accrued as the number of days from the index date until either the date of death or until right-censoring at the end of the study period (December 31, 2018), whichever occurred earlier. Likewise, follow-up time for ESKD accrued as the number of days from the index date until the date of incident ESKD or until right-censoring at the date of death or the end of the study period (December 31, 2018), whichever occurred earlier. Analyses for this study were performed from September 2022 to October 2023.

The baseline period was defined as the 2-year period prior to the index date of incident CKD. Homeless status (history of homelessness vs no history of homelessness), assessed during the baseline period only, was defined using data available to investigators with an algorithm adapted from service codes used to identify individuals in the VHA Homeless Services Registry and a previous publication.^12^ Service codes not explicitly indicated as a homeless service, such as inadequate housing, financial assistance for rent, or transitional housing, were not used. In brief, a veteran was categorized as having a history of homelessness in the presence of at least 1 service code of an outpatient or inpatient use of a VHA homeless service, or the presence of at least 1 *International Classification of Diseases, Ninth Revision (ICD-9)* or *International Statistical Classification of Diseases and Related Health Problems, Tenth Revision (ICD-10)* diagnosis code for homelessness (V60.0, Z59.0, or Z59.812).

Baseline demographic covariates included age (18-54, 55-64, 65-74, 75-84, and 85-99 years) at the index date, sex, and self-reported race and ethnicity, classified as Hispanic, non-Hispanic Black, non-Hispanic White, and non-Hispanic other or unknown. The non-Hispanic other category included veterans who identified as American Indian, Alaska Native, Asian, Native Hawaiian or Other Pacific Islander, or multiple races and were combined owing to small individual sample sizes. Additional covariates included eGFR at index date, smoking status (current, former, or never), body mass index (BMI; calculated as weight in kilograms divided by height in meters squared, as a continuous variable), medication use (angiotensin-converting-enzyme inhibitors, angiotensin II receptor blockers, and statins), and comorbidities. Comorbidities were assessed according to the presence of at least 1 *ICD-9* or *ICD-10* diagnosis code and included hypertension, depression, psychosis, anemia, obesity, alcohol use disorder, and substance use disorder, as well as those comprising the Charlson Comorbidity Index (CCI; with kidney disease excluded).^13^ Of note, diabetes is a component of the CCI. For both medication use and comorbidities, the absence of a record was assumed to represent a lack of history for the given medication or comorbidity. Veterans were excluded from the study if they were missing data on blood pressure measurements (as a proxy measure for regular VA use), smoking status, or BMI. These variables were extracted from records during the 2-year baseline period, with the exception of smoking status, which was based on the last VHA record prior to the index date.

### Statistical Analysis

Baseline characteristics were summarized by homeless status using counts and proportions for categorical variables, mean and SD for normally distributed continuous variables, and median and IQR for nonnormally distributed continuous variables. We obtained adjusted hazard ratios (HRs) and corresponding 95% CI to compare veterans with a history of homelessness with those without a history of homelessness. The association between homeless status and ESKD was examined using Cox regression models, with cause-specific HRs censored for death.^14^ The association between homeless status and death (including death after onset of ESKD) was examined using Cox regression models. We conducted 3 models adjusting for year of incident CKD and demographic characteristics. Model 1 included only year of incident CKD as a covariate, model 2 was further adjusted for age, and model 3 was further adjusted for sex and race and ethnicity. The proportional hazards assumption in model 3 was assessed by using the cox.zph function in R software. To evaluate the magnitude of variation in the HRs over time, we used a piecewise Cox model to estimate time-varying HRs across seven 2-year periods over the total 14 years of follow-up. To assess the role of additional variables that may be causes and/or intermediary factors for the association between homeless status and ESKD or death, 2 additional models were conducted in a secondary analysis. Model 4 was further adjusted for eGFR, BMI, CCI score, depression, hypertension, angiotensin-converting-enzyme inhibitor use, angiotensin II receptor blocker use, and statin use. Model 5 additionally was adjusted for smoking status, substance use disorder, alcohol use disorder, psychosis, and anemia. Exploratory analyses were conducted for each outcome by demographic subgroups (age group, sex, and race and ethnicity). Interaction tests were performed by testing for the statistical significance of interaction terms between homeless status and each of these demographic variables. SAS statistical software version 9.4 (SAS Institute) and R statistical software version 4.1.2 (R Project for Statistical Computing) were used to conduct statistical analyses. Two-sided *P* < .05 was considered statistically significant.

## Results

Among the 836 361 veterans with incident CKD included in the study, 46 561 (5.6%) had experienced homelessness (Table). The majority of veterans were male (809 584 veterans [96.8%]) and aged 65 to 74 years (274 371 veterans [32.8%]) or 75 to 84 years (270 890 veterans [32.4%]). A total of 26 037 veterans (3.1%) developed ESKD, and 359 991 (43.0%) died. Compared with veterans without a history of homelessness, those with a history of homelessness comprised a younger sample, a greater proportion of non-Hispanic Black veterans and current smokers, and had a higher prevalence of depression, alcohol use disorder, and substance use disorder. Event rates and follow-up time for each outcome, by homeless status, are shown in eTable 1 in Supplement 1.

**Table.** Baseline Characteristics of US Veterans With Incident Chronic Kidney Disease, by Homeless Status

Characteristic	Participants, No. (%)		
	Overall (N = 836 361)	History of homelessness (n = 46 561 [5.6%])	No history of homelessness (n = 789 800 [94.4%])
Age, y			
18-54	37 814 (4.5)	6545 (14.1)	31 269 (4.0)
55-64	152 801 (18.3)	17 890 (38.4)	134 911 (17.1)
65-74	274 371 (32.8)	12 665 (27.2)	261 706 (33.1)
75-84	270 890 (32.4)	7377 (15.8)	263 513 (33.4)
≥85	100 485 (12.0)	2084 (4.5)	98 401 (12.5)
Sex			
Female	26 777 (3.2)	2326 (5.0)	24 451 (3.1)
Male	809 584 (96.8)	44 235 (95.0)	765 349 (96.9)
Race and ethnicity			
Hispanic	31 276 (3.7)	2063 (4.4)	29 213 (3.7)
Non-Hispanic Black	133 588 (16.0)	17 759 (38.1)	115 829 (14.7)
Non-Hispanic White	593 926 (71.0)	24 291 (52.2)	569 635 (72.1)
Non-Hispanic other or unknown^a^	77 571 (9.3)	2448 (5.3)	75 123 (9.5)
Body mass index, mean (SD)^b^	29.7 (6.0)	29.7 (6.6)	29.7 (5.9)
Obesity^c^	214 463 (25.6)	14 036 (30.1)	200 427 (25.4)
Estimated glomerular filtration rate			
30-59 mL/min/1.73 m^2^	814 663 (97.4)	44 826 (96.3)	769 837 (97.5)
<30 mL/min/1.73 m^2^	21 698 (2.6)	1735 (3.7)	19 963 (2.5)
Smoking status			
Current	178 986 (21.4)	17 841 (38.3)	161 145 (20.4)
Former	466 423 (55.8)	20 859 (44.8)	445 564 (56.4)
Never	190 952 (22.8)	7861 (16.9)	183 091 (23.2)
Comorbidities			
Charlson Comorbidity Index score, mean (SD)	2.4 (2.1)	3.3 (2.6)	2.3 (2.1)
Depression	250 223 (29.9)	25 741 (55.3)	224 482 (28.4)
Psychosis	51 568 (6.2)	7259 (15.6)	44 309 (5.6)
Alcohol use disorder	72 170 (8.6)	14 408 (30.9)	57 762 (7.3)
Substance use disorder	46 662 (5.6)	14 133 (30.4)	32 529 (4.1)
Anemia	261 433 (31.3)	16 751 (36.0)	244 682 (31.0)
Hypertension	756 221 (90.4)	41 566 (89.3)	714 655 (90.5)
Medication use			
Angiotensin-converting enzyme inhibitors	460 029 (55.0)	28 257 (60.7)	431 772 (54.7)
Angiotensin II receptor blockers	111 009 (13.3)	5888 (12.6)	105 121 (13.3)
Statins	551 617 (66.0)	29 169 (62.6)	522 448 (66.1)

^a^ Other races included veterans who identified as American Indian, Alaska Native, Asian, Native Hawaiian or Other Pacific Islander, or multiple races.

^b^ Body mass index is calculated as weight in kilograms divided by height in meters squared.

^c^ Based on *International Classification of Diseases, Ninth Revision* and *International Statistical Classification of Diseases and Related Health Problems, Tenth Revision* codes.

**Figure 2.**
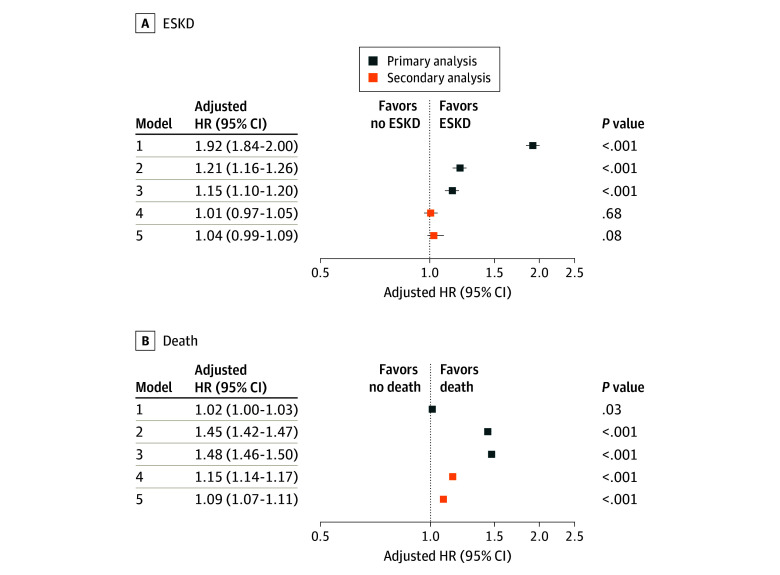
Association Between Homeless Status and End-Stage Kidney Disease (ESKD) or Death Among US Veterans Model 1 was adjusted for year of incident chronic kidney disease. Model 2 included model 1 plus adjustment for age. Model 3 included model 2 plus adjustment for sex and race and ethnicity. Model 4 included model 3 plus adjustment for estimated glomerular filtration rate, body mass index, obesity, Charlson Comorbidity Index, depression, psychosis, anemia, hypertension, angiotensin-converting-enzyme inhibitors use, angiotensin II receptor blocker use, and statin use. Model 5 included model 4 plus adjustment for smoking status, substance use disorder, and alcohol use disorder. Error bars denote 95% CIs. HR indicates hazard ratio.

Figure 2 displays adjusted HRs for the association between homeless status and ESKD or death. After adjustment for year of incident CKD (model 1), homelessness was significantly associated with a nearly 2-fold increase in ESKD risk (HR, 1.92; 95% CI, 1.84-2.00). After additional adjustment for age (model 2), the HR attenuated to 1.21 (95% CI, 1.16-1.26), but further adjustment for sex and race and ethnicity (model 3) did not substantially change the results (HR, 1.15; 95% CI, 1.10-1.20). Although homeless status was not significantly associated with death in model 1 (HR, 1.02; 95% CI, 1.00-1.03), results became significant after adjustment for age in model 2 (HR, 1.45; 95% CI, 1.42-1.47). In model 3, results were similar (HR, 1.48; 95% CI, 1.46-1.50) to that in model 2. Testing for the proportional hazards assumption in model 3 suggested that the proportional hazards assumption was violated. In the subsequent analysis of time-varying HRs, the association between homeless status and both ESKD and death attenuated over time (eTable 2 in Supplement 1). Specifically, the increased risk of ESKD associated with homeless status was higher in earlier years of follow-up and was no longer significant in later years. The increased risk of death followed a similar pattern, although it remained statistically significant throughout the entire follow-up period.

In secondary analyses adjusting for additional factors (eg, eGFR, BMI, comorbidities, medication use, and smoking status) that may be confounders and/or mediators, homeless status was no longer significantly associated with incident ESKD (model 4, HR, 1.01; 95% CI, 0.97-1.05; model 5, HR, 1.04; 95% CI, 0.99-1.09). In the analogous secondary analyses for all-cause death, HRs were attenuated but remained significant for both model 4 (HR, 1.15; 95% CI, 1.14-1.17) and model 5 (HR, 1.09; 95% CI, 1.07-1.11).

**Figure 3.**
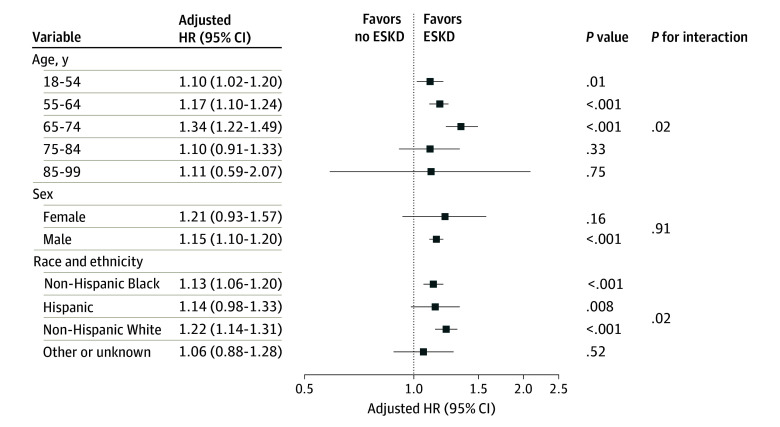
Association Between Homeless Status and End-Stage Kidney Disease (ESKD) Among US Veterans, by Demographic Subgroups Hazard ratios (HRs) represent the risk of ESKD among veterans experiencing homelessness compared with those not experiencing homelessness. Models are adjusted for year of incident chronic kidney disease, age, sex, and race and ethnicity and exclude the demographics used as the stratification variables. Error bars denote 95% CIs.

Adjusted HRs for the association between homeless status and ESKD among demographic subgroups are shown in Figure 3. Among subgroups of age, the largest HR of ESKD for veterans with a history of homelessness compared with those without a history of homelessness was among veterans aged 65 to 74 years (HR, 1.34; 95% CI, 1.22-1.49). HRs were more attenuated among other age groups, and wide 95% CIs were observed among older age groups owing to fewer cases of ESKD in this group. An overall test of significance for interaction between homeless status and age group showed statistical significance (*P* for interaction, .02). Among subgroups of sex, similar HRs were observed for both female and male veterans, although the HR was statistically significant for male veterans only (HR, 1.15; 95% CI, 1.10-1.20), owing to a much smaller sample size for female veterans. Among subgroups of race and ethnicity, point estimates for HRs were similar across all groups, with statistically significant HRs for non-Hispanic Black (HR, 1.13; 95% CI, 1.06-1.20) and non-Hispanic White (HR, 1.22; 95% CI, 1.14-1.31) veterans (overall *P* for interaction, .02).

**Figure 4.**
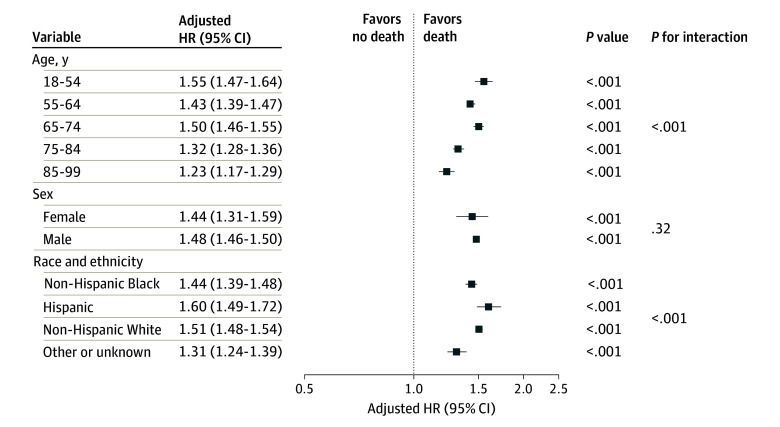
Association Between Homeless Status and Death Among US Veterans, by Demographic Subgroups Hazard ratios (HRs) represent the risk of death among veterans experiencing homelessness compared with those not experiencing homelessness. HRs are adjusted for year of incident chronic kidney disease, age, sex, and race and ethnicity and exclude the demographics used as the stratification variables. Error bars denote 95% CIs.

Figure 4 shows adjusted HRs for the association between homeless status and death, by demographic subgroups. Among all subgroups (age, sex, and race and ethnicity), veterans with a history of homelessness showed a significantly greater risk of death vs veterans without a history of homelessness. Among all age groups, the youngest veterans aged 18 to 54 years showed the largest HR (1.55; 95% CI, 1.47-1.64). An overall test of significance for the interaction between homeless status and age was statistically significant (*P* for interaction, <.001). HRs among female (HR, 1.44; 95% CI, 1.31-1.59) and male (HR, 1.48; 95% CI, 1.46-1.50) veterans were similar. Among subgroups of race and ethnicity, Hispanic veterans showed the largest HR (HR, 1.60; 95% CI, 1.49-1.72; overall *P* for interaction, <.001).

## Discussion

In this cohort study of a large nationwide population of veterans with incident CKD, we observed a greater risk of both ESKD and death among veterans with a history of homelessness compared with those who did not have a history of homelessness. Results from secondary analyses suggest that particularly for ESKD, the greater risk may be attributed to a greater comorbidity burden among veterans with a history of homelessness. In analyses exploring time-varying HRs, results were more attenuated in later years of follow-up, particularly for incident ESKD, which did not show significant results after the first 6 years of follow-up. In subgroup analyses evaluating risk of ESKD or death, results differed most substantially by subgroups of age or race and ethnicity for the risk of death.

Few prior studies have evaluated the role of homelessness in the risk of ESKD or death among adults with CKD. Two prior studies^3,4^ comprising the same source population from San Francisco, California, studied approximately 15 000 uninsured adults with CKD. The authors found that homelessness was significantly associated with a 1.8-fold greater risk of either ESKD or death, in a crude analysis, and with a 1.28-fold greater risk after adjustment for sociodemographic factors, comorbidities, and other clinical characteristics.^3,4^ Although these findings slightly differ from those of the current study, the sociodemographic and clinical characteristics of the VHA population in the current study differed. For example, the VHA population comprised a greater proportion of male individuals and non-Hispanic White adults and a higher prevalence of comorbidities, such as hypertension and depression. Outside the US, studies in Ghana^6^ and Mexico^7^ have evaluated CKD among adults experiencing homelessness, although those studies reported limited cross-sectional findings, such as prevalence estimates for CKD,^6^ and lack of awareness of CKD and associated comorbidities, such as diabetes and hypertension.^7^

Although clinical characteristics of the cohort showed substantial differences by homeless status, only some of these factors, such as age and comorbidities, appeared to be greatly associated with risk of ESKD or death. Adjusting for comorbidities substantially attenuated HRs, suggesting that among all evaluated risk factors, they may contribute the most to risk of ESKD or death. These results are consistent with the established role of cardiovascular disease and diabetes as risk factors for progression to ESKD^15,16^ and a high risk of cardiovascular death among adults with CKD.^17^ Compared with veterans without a history of homelessness, those with a history of homelessness had a substantially higher prevalence of current smoking and substance use disorder, which may be associated with an increased risk of ESKD or death.^18,19,20^ However, further adjustment for these factors, after accounting for comorbidities, did not substantially affect the results, suggesting that other comorbidities may be greater contributors to risk of ESKD and death in this population. Alternatively, the potential associations of substance use with ESKD and death may have been already accounted for in the regression model after adjustment for other comorbidities.

The association between homeless status and risk of ESKD or death did not substantially differ after adjustment for sex and race or ethnicity, although the lack of changes in the HR for the former factor may be because of the predominance of male veterans in the sample. Results also did not substantially differ among most demographic subgroups. However, prior studies among adults with CKD have shown that sex and race or ethnicity may be determinants of health outcomes. For example, prior evidence suggests a greater risk of progression to ESKD among men compared with women^21,22^ and the highest risk of progression of CKD to ESKD among American Indian/Alaska Native, Asian, Black, Hispanic, and Pacific Islander adults.^23,24^ The lack of a major role for sex and race and ethnicity may be attributed to the substantial impact of a history of homelessness on health outcomes that may override the potential impact of other demographic factors. Individuals experiencing homelessness represent the most socioeconomically disadvantaged population in terms of income, housing, health care access, air pollution exposure, climate change, and other related social determinants of health.^25,26,27^ Health outcomes of adults with CKD experiencing homelessness may, therefore, be most affected by homelessness itself, underscoring the role of housing as a fundamental prerequisite for sufficient management of chronic disease.^28,29^

Follow-up care is frequently required for proper management of CKD,^30^ but maintenance of regular care is likely particularly challenging among adults experiencing homelessness. To ensure sufficient health care for veterans experiencing homelessness, effective and tailored methods may be needed. For example, McGuire et al^31^ reported that providing homeless, primary care, and mental health services at the same location resulted in a more than 2-fold increase in primary care visits over an 18-month follow-up period compared with a group receiving geographically separate primary care and homeless services. O’Toole and colleagues^32^ conducted an early pilot study of the Homeless Patient Aligned Care Team treatment model designed to provide veterans experiencing homelessness with increased access to primary care and to prevent adverse health outcomes.^33^ In that study, investigators conducted an intervention consisting of outreach plus clinic orientation for veterans experiencing homelessness who were not receiving any primary care at the time of enrollment and found at 6 months that 89% of participants accessed primary care compared with only 37% in the usual care group.^32^ Additional studies^34^ assessing the Homeless Patient Aligned Care Team model have generally reported favorable outcomes, such as increased usage of primary care and patients reporting positive experiences with health care access, although the quality of the evidence base is limited to observational studies, and it is unknown whether the program can increase access to housing. To maximize effectiveness, future interventions may need to comprehensively address the many barriers to health care access faced by veterans experiencing homelessness. These barriers include distrust in health care services, feelings of judgment or stigma from practitioners, prioritizing basic needs such as food and shelter over health care, lack of transportation, and lack of knowledge of available health care resources.^35,36^

### Strengths and Limitations

Strengths of the current study include the large nationwide cohort with available data on clinical diagnoses, pharmaceutical treatment, and the use of an incident CKD cohort at baseline, which may reduce confounding by disease stage. Moreover, the use of both VHA records and Medicare claims to measure comorbidities likely provides greater validity than using VHA data alone, as is commonly done in other studies.

Limitations include residual confounding from imprecise measurement of confounders or unmeasured confounders that may have affected results. For example, although depression, alcohol use disorder, and substance use disorder were adjusted for in model 5, other psychiatric comorbidities may also be confounders and/or intermediaries. Although the homelessness spectrum ranges from long-term unsheltered living to isolated periods of temporary housing, we did not have sufficient data to accurately characterize homelessness at more granular levels of detail or as a time-varying variable. Use of VHA homeless services and *ICD-9* or *ICD-10* codes for homelessness may capture individuals across this spectrum. However, we used a multisource method to identify veterans experiencing homelessness, which has been demonstrated to capture the majority of documented cases of homelessness in the VHA.^37^ Because our data were derived from a cohort of predominantly male veterans, these results may not be generalizable to the adult population experiencing homelessness in the US. In particular, the study might have selected a care-seeking subset of veterans with a history of homelessness, which might have underestimated the risk of ESKD and death in the general population of adults with a history of homelessness, who may seek less care and not be eligible to utilize VHA services.

## Conclusions

This study demonstrated the high risk of adverse health outcomes among veterans with CKD with a history of homelessness, despite comprising a population with access to both health insurance and programs to provide social, financial, and other forms of assistance to individuals experiencing homelessness. Potential interventions to improve CKD management may include tailored outreach to increase access to care in this population. Long-term solutions may require recognition of housing as a primary determinant of health and prioritization of housing to ensure adequate management of CKD and any other chronic conditions.

## Notes

Supplement 1. **eTable 1.** Event Rates and Follow-up Time for US Veterans with Incident Chronic Kidney Disease, by Homeless Status **eTable 2.** Time-Varying Hazard Ratios for the Association Between Homeless Status and End-Stage Kidney Disease or Death Among US Veterans

Supplement 2.
Data Sharing Statement
