# Supplementary material for: Homelessness and Risk of End-Stage Kidney Disease and Death in Veterans With Chronic Kidney Disease
Source: JAMA Netw Open. 2024 Sep 10;7(9):e2431973. doi: 10.1001/jamanetworkopen.2024.31973 (PMC11388027; doi:10.1001/jamanetworkopen.2024.31973)
Supplement: Supplement 1. — eTable 1. Event Rates and Follow-up Time for US Veterans with Incident Chronic Kidney Disease, by Homeless Status eTable 2. Time-Varying Hazard Ratios for the Association Between Homeless Status and End-Stage Kidney Disease or Death Among US Veterans [file jamanetwopen-e2431973-s001.pdf]

Supplemental Online Content

Koyama AK, Nee R, Yu W, et al. Homelessness and risk of end-stage kidney disease and death in veterans with chronic kidney disease. *JAMA Netw Open*. 2024;7(9):e2431973. doi:10.1001/jamanetworkopen.2024.31973

eTable 1. Event Rates and Follow-up Time for US Veterans with Incident Chronic Kidney Disease, by Homeless Status

eTable 2. Time-Varying Hazard Ratios for the Association Between Homeless Status and End-Stage Kidney Disease or Death Among US Veterans

This supplemental material has been provided by the authors to give readers additional information about their work.

© 2024 Koyama AK et al. *JAMA Network Open*.

eTable 1. Event Rates and Follow-up Time for US Veterans with Incident Chronic Kidney Disease, by Homeless Status

Outcome	History of Homelessness	Number of Events (percent)	Event rate (per 1,000 patient-years)	Years of Follow-Up, Median (IQR)
Incident ESKD	No	23,587 / 789,800 (3.0%)	6.0	4.2 (2.2-7.4)
	Yes	2,450 / 46,561 (5.3%)	11.3	4.0 (2.0-6.8)
All-Cause Death	No	340,878 / 789,800 (43.2%)	84.9	4.3 (2.3-7.5)
	Yes	19,113 / 46,561 (41.0%)	85.3	4.1 (2.1-7.0)

Abbreviations: IQR = Interquartile Range; ESKD, end-stage kidney disease

eTable 2. Time-Varying Hazard Ratios for the Association Between Homeless Status and End-Stage Kidney Disease or Death Among US Veterans

2-year interval from index date	Hazard Ratio (95% CI)	
	ESKD	Death
0-2	1.31 (1.22-1.41)	1.79 (1.74-1.83)
2-4	1.16 (1.07-1.26)	1.45 (1.41-1.50)
4-6	1.14 (1.04-1.26)	1.35 (1.31-1.40)
6-8	0.95 (0.83-1.08)	1.30 (1.25-1.36)
8-10	0.92 (0.77-1.11)	1.23 (1.16-1.30)
10-12	0.86 (0.65-1.14)	1.21 (1.12-1.31)
12-14	1.01 (0.61-1.68)	1.15 (1.00-1.32)

Hazard ratios were adjusted for age, sex, race and ethnicity, and year of incident chronic kidney disease

Abbreviations: CI = confidence interval; ESKD, end-stage kidney disease

© 2024 Koyama AK et al. *JAMA Network Open*.
