# Supplementary material for: Homelessness and Risk of End-Stage Kidney Disease and Death in Veterans With Chronic Kidney Disease
Source: JAMA Netw Open. 2024 Sep 10;7(9):e2431973. doi: 10.1001/jamanetworkopen.2024.31973 (PMC11388027; doi:10.1001/jamanetworkopen.2024.31973)
Supplement: Supplement 2. — Data Sharing Statement [file jamanetwopen-e2431973-s002.pdf]

# Data Sharing Statement

Koyama. Homelessness and Risk of End-Stage Kidney Disease and Death in Veterans With Chronic Kidney Disease. *JAMA Netw Open*. Published September 10, 2024.
doi:10.1001/jamanetworkopen.2024.31973

**Data**
**Data available:** No

**Additional Information**
**Explanation for why data not available:** Per the terms of the data use agreement with the Veterans Health Administration, data cannot be made publicly available.
